# Quality of Drinking Water Treated at Point of Use in Residential Healthcare Facilities for the Elderly

**DOI:** 10.3390/ijerph120911163

**Published:** 2015-09-09

**Authors:** Rossella Sacchetti, Giovanna De Luca, Emilia Guberti, Franca Zanetti

**Affiliations:** 1 Department of Education Sciences, Hygiene, University of Bologna, Via San Giacomo 12, 40126 Bologna, Italy; 2 Department of Biomedical and Neuromotor Sciences, Unit of Hygiene, Public Health and Medical Statistics, University of Bologna, Via San Giacomo 12, 40126 Bologna, Italy; E-Mails: giovanna.deluca@unibo.it (G.D.L.); franca.zanetti@unibo.it (F.Z.); 3 Department of Public Health, UO Food Hygiene and Nutrition, Local Health Unit of Bologna, Via Altura 5, 40100 Bologna, Italy; E-Mail: emilia.guberti@ausl.bologna.it

**Keywords:** drinking water, point-of-use devices, healthcare facilities for the elderly, opportunistic pathogens, mineral content of drinking water

## Abstract

Municipal tap water is increasingly treated at the point of use (POU) to improve the acceptability and palatability of its taste. The aim of this study was to assess the bacteriologic and nutritional characteristics of tap water treated at the point of use in residential healthcare facilities for the elderly. Two types of POU devices were used: microfiltered water dispensers (MWDs) and reverse-osmosis water dispensers (ROWDs). All samples of water entering the devices and leaving them were tested for the bacteriological parameters set by Italian regulations for drinking water and for opportunistic pathogens associated with various infections in healthcare settings; in addition, the degree of mineralization of the water was assessed. The results revealed widespread bacterial contamination in the POU treatment devices, particularly from potentially pathogenic species. As expected, the use of ROWDs led to a decrease in the saline content of the water. In conclusion, the use of POU treatment in healthcare facilities for the elderly can be considered advisable only if the devices are constantly and carefully maintained.

## 1. Introduction

On the basis of extensive data systematically collected both at a local and a national level, it can be asserted that the municipal tap water supplied in Italy is qualitatively suitable for human consumption and is controlled through an efficient surveillance system [1]. Italian regulations for drinking water (Decreto Legislativo 31/2001, the application of EC Directive 98/83) set standards for public water supplies that limit the levels of contaminants, and regular tests are carried out to ensure that these standards are met [2,3].

However, many people continue to be reluctant to drink tap water, and the use of “refinement treatments” is becoming increasingly common in order to obtain a more acceptable and palatable taste, both in domestic settings and in public establishments (restaurants, schools, offices, *etc*.). A recent survey shows that in Italy, 36.6% of those who habitually drink tap water use a drinking water treatment device [4]. Numerous types of devices that treat the water at the point of use (POU devices) are commercially available, making use of different refinement treatments [4]. Such devices are marketed as being able to eliminate unpleasant odors and tastes and to remove any undesirable substances from the tap water. They often include systems for the addition of CO_2_ and for the cooling of the water. Compared to bottled water, these devices offer the advantage of avoiding the need for the transport, storage and disposal of the bottles.

However, the most common drawback is bacterial growth, which was detected in microfiltering devices [5,6,7]. Some of the bacteria found in water dispensed by these devices are associated with infections in healthcare settings [8,9].

People living in healthcare facilities for the elderly are generally very vulnerable due to their advanced age and the presence of chronic pathologies. It is important that the drinking water used in these settings be safe from a microbiological point of view and also satisfactory from a nutritional point of view.

In the present study, we investigated the bacteriological quality of municipal tap water treated by different kinds of POU water treatment systems in healthcare facilities for the elderly; in particular, the occurrence and concentration of some opportunistic pathogens was determined. In addition, some physical and chemical characteristics regarding the degree of mineralization of the water were assessed.

## 2. Experimental Section

### 2.1. Point of Use Water Treatment Devices

The study examined devices that treat municipal tap water at the point of use in healthcare facilities for the elderly in the area of Bologna, Northern Italy. All facilities are long-term care homes for frail elderly people with physical and/or mental age-related diseases. When the study was carried out, only 19 establishments used POU water treatment systems, for a total of 38 units. Two types of devices were in use:

▪ Microfiltered water dispensers (MWDs) (*n* = 20) with composite filters (EVERPURE). The filters consist of a disposable cartridge containing a membrane (0.5-micron pore size) made of polyethylene fibers and powdered activated carbon. The single-use cartridges are replaced once a year, and the circuits are disinfected twice a year with a stabilized aqueous solution of hydrogen peroxide. The devices examined had been in use for a mean of 41 months and dispensed around 35 L of water a day.
▪ Reverse-osmosis water dispensers (ROWDs) (*n* = 18) with a sediment pre-filter, an activated carbon filter, an RO membrane (rated at 4 L/h), a 19 L storage tank, an activated carbon post-filter and a UV lamp. The filters (sediment pre-filter and 2 activated carbon filters) are replaced once a year, and the tank and the tubes are descaled and disinfected (with a multicomponent product based on acids and a stabilized aqueous solution of hydrogen peroxide) once a year. In addition, the devices all have a bypass mixer valve, whose function is to regulate the saline content of the dispensed water. The devices had a mean age of 71 months and dispensed around 35 L of water a day.

All of the devices were directly attached to the municipal water supply and dispensed still unchilled water and still chilled water.

The age of use of each device and the number of elderly people served are shown in Table 1.

**Table 1:** Description of some characteristics of the devices in the residential healthcare facilities for the elderly.

Healthcare Facilities for the Elderly		Kind of Devices	Number of Devices	Number of Individuals Served	Age of Devices (in Months)
1	C.M.	MWD	4	102	54
2	L.U.	MWD	3	58	36, 36, 13
3	S.N.	MWD	2	25	12, 8
4	M.	MWD	1	20	20
5	V.C.	MWD	1	22	120
6	G.	MWD	1	10	8
7	A.	MWD	2	50	29
8	F.	MWD	1	31	101
9	S.G.	MWD	1	22	41
10	M.T.	MWD	1	15	10
11	R.M.	MWD	1	18	45
12	B.	MWD	2	32	41, 50
13	V.O.	ROWD	1	47	119
14	S.	ROWD	4	82	108
15	P.	ROWD	5	45	12
16	R.A.	ROWD	3	100	98
17	R.	ROWD	1	21	98
18	V.F.	ROWD	1	20	54
19	S.B.	ROWD	3	60	41, 91, 91

MWD = microfiltered water dispenser; ROWD = reverse osmosis water dispenser.

### 2.2. Sample Collection

Each sampling session involved the simultaneous collection of:

▪ Two samples of water from each device, 1 still unchilled water and 1 still chilled water.
▪ One sample of municipal tap water entering the dispenser (generally from the nearest tap to the device).

A total of 114 water samples were analyzed, consisting of 38 samples of municipal tap water and 76 samples of POU-treated water (20 still unchilled water and 20 still chilled water from MWDs, 18 still unchilled water and 18 still chilled water samples from ROWDs).

In order to assess the types of bacteria the consumer actually ingests from the tap water, the taps were not flamed or sanitized before sample collection, as in previous studies [7,10,11].

The samples were always taken in the morning, after the devices had been working for about an hour. For bacteriological analyses, 1 L samples were collected in sterile plastic bottles containing 1 mL of a sterile sodium thiosulfate solution (10% w/v) to neutralize any residual chlorine. For chemical analyses, 1 L samples were collected in plastic bottles. All samples were then stored in a plastic cooler and packed with ice for transport to the laboratory for immediate processing.

### 2.3. Bacteriological Analyses

Bacteriological parameters detected in water samples are shown in Table 2. In accordance with Italian regulations for drinking water [2], they were enumerated for each sample: *Escherichia coli* (EC), enterococci (ENT), total coliforms (TC)*,* heterotrophic plate count at 22 °C (HPC 22 °C), *Pseudomonas aeruginosa* (PA) and *Staphylococcus aureus* (SA). In the Italian regulation, PA and SA are “supplementary” parameters to be determined at the discretion of the local health authority.

The microbial criteria for unbottled municipal tap water established by the law are shown in Table 2, as well. Italian regulations set no numerical value for HPC at 22 °C, but state that there should be no “abnormal changes” compared to the values obtained during routine official checks. No specific limit is set for PA.

**Table 2:** Bacteriological parameters determinated in water samples.

Bacteriological Parameters	Microbial Criteria for Unbottled Water (Italian Regulation for Drinking Water)
*Escherichia coli* (EC)	0/100 mL
Enterococci (ENT)	0/100 mL
Total coliforms (TC)	0/100 mL
Heterotrophic plate count (HPC) 22 °C	“no abnormal change”
Heterotrophic plate count (HPC) 37 °C *	
*Staphylococcus aureus* (SA)	0/250 mL
*Pseudomonas aeruginosa* (PA) and other non-fermentative Gram-negative bacteria (NF-GNB) **	
Coagulase-negative staphylococci (CoNS) **	

* The determination of this parameter is not required for unbottled drinking water by the Italian regulation. ** The determination of these parameters is not required by the Italian regulation for drinking water; NF, non -fermentative.

HPC at 37 °C was also determined to obtain a more complete assessment of the bacteriological quality of the water in question. In Italy, the measurement of the HPC at 37 °C is required only for water sold in bottles or containers. Furthermore, we determined the occurrence and concentration of other species of non-fermentative Gram-negative bacteria (NF-GNB) and coagulase-negative staphylococci (CoNS).

Bacterial analyses were performed according to the Standard Methods for the Examination of Water and Wastewater [12]. HPC bacteria at 22 °C and 37 °C were enumerated by the pour plate method using plate count agar (Oxoid, Milan, Italy). The mean value of three replicates was calculated. The detection limit was 1 cfu/mL.

The membrane filtration technique using 0.45-micron pore size filters (Millipore, Milan, Italy) was used for the enumeration of EC, ENT, TC, PA, non-fermentative Gram-negative bacteria (NF-GNB), SA and coagulase-negative staphylococci (CoNS), as listed below. One-hundred-milliliter water samples were used to enumerate EC, ENT and CT; 250-mL water samples were tested for PA, NF-GNB, SA and CoNS. The detection limit was 1 cfu per sample volume for all types of bacteria.

EC: The filter was transferred to C-EC agar (Biolife Milan, Italy). After incubation at 44.5 °C for 24 h, typical colonies (fluorescent green-blue under a Wood lamp and positive to the indole test) were counted. Doubtful colonies underwent biochemical identification using the Enterotube II system (BBL- Becton Dickinson, Milan, Italy).

ENT: The filter was transferred to Enterococcus agar (Oxoid). After incubation at 35 °C for 24–48 h, typical colonies (pink-brown in color and 0.3–2 mm in diameter) were confirmed by growth on bile aesculin agar (Oxoid) at 35 °C for 48 h and by growth on brain-heart infusion broth (Oxoid) with 6.5% NaCl at 35 °C for 48 h.

TC: The filter was transferred to CEC agar (Biolife). After incubation at 37 °C for 24 h, typical colonies (green-blue) were counted. Doubtful colonies underwent biochemical identification using the Enterotube II system (BBL).

PA and other NF-GNB: The filters were placed on Pseudomonas CFC agar (Oxoid) and incubated at 30 °C for 24*–*48 h. Colonies that were smooth, mucoid, fluorescent, blue-green or yellow-green in color, with diffuse pigmentation of the medium, were presumed to be *P. aeruginosa*. They were subsequently subcultured on tryptone soya agar (TSA-Oxoid) and identified by the API 20NE system (BioMérieux, Marcy l’Etoile, France). The other colonies were also counted, and at least 5 colonies per plate, or all if less than 5, were subcultured on TSA (Oxoid) and identified by the API 20NE system (BioMérieux).

SA and CoNS*:* The filter was incubated in Staph 110 medium (Oxoid) at 36 °C for 40–48 h. All presumed colonies of *S. aureus* (dark orange pigmented) and all of the non-pigmented colonies (white and yellow) were differentiated and counted; at least 5 colonies of each type, or all if less than 5, were sub-cultured on TSA (Oxoid). The API Staph System (BioMérieux) was used for the identification.

### 2.4. Physical and Chemical Analyses

All water samples were tested for temperature and residual chlorine at the time of collection, respectively with a mercury thermometer and the DPD (*N*,*N*-diethyl-p-phenylenediamine) colorimetric method [12]. In addition, measurements were made of the pH (potentiometric method) [12], the electric conductivity [12], the concentration of calcium and sodium (ICP-OES method) [13], total water hardness [12] and total dissolved solids at 180 °C (gravimetric method) [12].

### 2.5. Statistical Analysis

The values of microbial concentrations were converted into log_10_ colony-forming units (log_10_ cfu). For all negative samples, the detection limits were used.

The variation in the HPCs of the input and output water was calculated using the following formula: [(log_10_ B –log_10_ A)/log_10_ A], where A = HPC in the input water and B = HPC in the output water. The HPC values were expressed in log_10_ (*x* + 1). Differences were considered significant as determined by ANOVA. Correlations were determined between variations in HPC and residual chlorine and between variations in HPC and the age of the device.

The significance level chosen for all analyses was *p* < 0.05. Analyses were performed using the StatView program (Abacus Concepts Inc., Berkley, CA, USA) on an Apple Macintosh computer.

## 3. Results

### 3.1. Bacteriological Characteristics of the Water

The results of the HPCs for the water samples are presented in Table 3. The mean HPCs at 22 °C and 37 °C were significantly higher in output water samples compared to those of the input water (*p* < 0.05).

**Table 3:** Mean values and standard deviation of HPCs.

	22 °C HPC (Log cfu/mL)		37 °C HPC (Log cfu/mL)	
	Mean	SD	Mean	SD
MWDs				
municipal tap water	0.53	0.48	0.95	0.53
still unchilled water	0.91	0.54	1.57	0.54
still chilled water	0.71	0.51	1.37	0.50
ROWDs				
municipal tap water	0.42	0.48	0.74	0.32
still unchilled water	1.45	0.46	1.58	0.52
still chilled water	1.44	0.50	1.32	0.59

**Figure 1:**
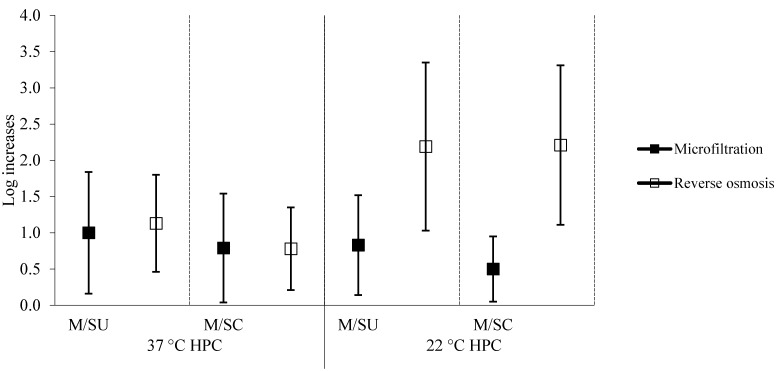
Average log increases (bars showing the 95% confidence intervals) of the HPCs. M = municipal tap water; SU = still unchilled water; SC = still chilled water.

The average variations of HPCs at 22 °C and 37 °C in POU microfiltered/RO-treated water *vs*. municipal tap water are shown in Figure 1.

The highest average increase (log 2.2) was observed in HPCs at 22 °C in the water subjected to reverse osmosis; only the increase in HPCs at 22 °C in the chilled RO-treated water was significantly higher than the increase registered in microfiltered chilled water samples (*p* < 0.05).

The variations in HPCs at 22 °C in the water subjected to reverse osmosis was directly correlated with the age of the devices (*p* < 0.005).

EC was consistently absent. ENT were detected in one unchilled sample and in one chilled sample dispensed by ROWDs (at concentrations of, respectively, 1 and 2 cfu/100 mL). TC were found in one tap water sample (5 cfu/100 mL) and in one microfiltered unchilled water sample (1 cfu/100 mL).

The frequencies of samples positive for NF-GNB and staphylococci and the various species isolated are shown in Table 4. *P. aeruginosa*, detected in around 10% of samples of input water, was found more frequently and at higher concentrations in the samples of water dispensed by the MWDs. In the water entering and leaving the MDWs, another 11 species of NF-GNB were found, the most interesting from a health point of view being *S. maltophilia*, *P. putida* and *D. acidovorans*. In the water entering and leaving the RODWs, *D. acidovorans* and *B. pseudomallei* are worthy of note. *Staphylococcus aureus* was detected only once, in a sample of input water. The highest number of CoNS species was found in the microfiltered water.

### 3.2. Physical and Chemical Characteristics of the Water

A summary of the physical and chemical data for the water samples is shown in Table 5. The temperature of the still unchilled water samples was on average higher than that of tap water samples. Moreover, the levels of residual chlorine in the POU microfiltered/RO-treated water samples were halved compared to those of tap water samples.

Microfiltration treatment does not determine any notable variations in the values of the parameters for mineralization, whereas the reverse osmosis treatment substantially reduces the sodium and calcium content, as well as the values of electric conductivity, hardness, total dissolved solids at 180 °C and pH.

A negative trend was observed between the variations in HPC values and the residual chlorine present in the water, although these correlations were not statistically significant.

**Table 4:** Prevalence of NF-GNB and staphylococci species and the range of isolates.

	NF-GNB			Staphylococci (SA + CoNS)		
	Species	Number of Positive Samples	Range (cfu/250 mL)	Species	Number of Positive Samples	Range (cfu/250 mL)
						
**MWDs**						
M	*P. aeruginosa*	2	1–2.	*S. aureus*	1	1
	*D. acidovorans*	1	2	*S. epidermidis*	1	1
	*Alc. xylosoxidans*	1	4	*S. haemolyticus*	1	1
	*C. testosteroni*	1	50	*S. hominis*	6	1–1.
	*P. putida*	1	2	*S. saprophyticus*	1	2
SU	*P. aeruginosa*	5	19–550	*S. epidermidis*	1	1
	*P. aureofaciens*	1	24	*S. haemolyticus*	1	1
	*P. fluorescens*	2	5–7.	*S. hominis*	1	1
	*Moraxella* spp.	1	1	*S. saprophyticus*	1	1
	*P. putida*	2	2–28.	*S. xylosus*	2	1–1.
	*S. maltophilia*	1	9			
SC	*P. aeruginosa*	7	1–573	*S. hominis*	2	1–1.
	*P. pickettii*	1	22	*S. saprophyticus*	1	1
	*P. stutzeri*	1	64	*S. warneri*	1	2
	*S. maltophilia*	2	10–1350			
**ROWDs**						
M	*P. aeruginosa*	2	1–19.	*S. conhii*	1	2
	*D. acidovorans*	1	633			
	*P. aureofaciens*	1	4			
	*Moraxella* spp.	1	850			
	*P. stutzeri*	1	1			
SU	*P. aureofaciens*	1	5			
	*P. fluorescens*	1	2			
SC	*P. aeruginosa*	3	4–10.	*S. conhii*	2	1–2.
	*P. aureofaciens*	4	1–298	*S. epidermidis*	1	1
	*P. fluorescens*	1	2	*S. haemolyticus*	2	1–3.
	*B. pseudomallei*	1	16	*S. warneri*	1	34

M = municipal tap water; SU = still unchilled water; SC = still chilled water.

**Table 5:** Mean values and standard deviation of physical-chemical parameters.

	MWDs		ROWDs	
	mean	SD	Mean	SD
Water temperature (°C)				
municipal tap water	17.7	3.8	15.0	4.8
still unchilled water	21.8	4.0	17.8	3.5
still chilled water	7.9	3.9	8.2	2.6
Residual chlorine (mg/L)				
municipal tap water	0.14	0.11	0.22	0.10
still unchilled water	0.07	0.07	0.10	0.10
still chilled water	0.06	0.06	0.10	0.10
pH value				
municipal tap water	7.42	0.33	8.07	0.17
still unchilled water	7.40	0.27	6.87	0.44
still chilled water	7.42	0.27	6.83	0.42
Conductivity (mS/cm)				
municipal tap water	600	157	493	52
still unchilled water	602	153	69	63
still chilled water	597	150	67	62
Calcium (mg/L)				
municipal tap water	93.0	28.1	71.9	7.5
still unchilled water	90.8	26.7	7.0	8.9
still chilled water	92.4	30.8	6.1	7.2
Sodium (mg/L)				
municipal tap water	26.6	9.0	20.4	4.4
still unchilled water	26.2	11.5	4.0	3.4
still chilled water	25.5	8.2	3.8	3.7
Total hardness (°F)				
municipal tap water	29.1	8.9	23.5	2.9
still unchilled water	28.8	8.6	2.3	3.0
still chilled water	29.7	10.3	2.4	2.7
Total dissolved solids at 180 °C (mg/L)				
municipal tap water	406	115	311	45
still unchilled water	409	114	43	38
still chilled water	404	111	46	36

## 4. Discussion

Overall, 96% of the water samples were found to comply with the bacteriological standards for unbottled drinking water. EC was consistently absent, while ENT were detected in two samples of water dispensed by the ROWDs. It is likely that the presence of ENT in the RO-treated water derives from a contamination of the water distribution points by residents and staff of the healthcare establishment.

TC were detected in two samples. TC are defined as an “indicator” parameter of the water quality [2], and therefore, the isolation of a few units of TC does not, at least in the first instance, require the competent health authorities to take corrective and precautionary measures to safeguard the population. Some species of TC can, in fact, have an environmental origin (*Serratia liquefaciens*, *Serratia marcescens*, *Klebsiella terrigena*) and are able to multiply in water and can also grow in water distribution systems, particularly in the presence of biofilm [14]. Moreover, in this case, also, the contamination of the distribution point cannot be ruled out.

Although current Italian regulations do not stipulate any limit for HPCs, the values found in this study were on average higher in the water dispensed by POU devices than in municipal tap water. The increase in HPC values could be related to the formation of a microbial biofilm on the surfaces of the devices in contact with the water [15]. The biofilm appears as three-dimensional functional consortia of microbial cells, bound to and growing at an interface enveloped within extracellular polymers [16].

The highest increases in HPC were found in the water leaving the ROWDs. Reverse osmosis membranes are known to be able to retain more than 99% of the bacterial cells present in tap water [14]. Park and Hu reported that remarkable biofilm accumulation and bulk cell growth occurred in RO permeate water flowing through a model distribution system [17]. The RO devices examined in our study had a higher mean age than MWDs and included a storage tank to collect permeate water. Water stagnation is well documented as favoring bacterial regrowth and the formation of biofilm [18]. The higher increases in HPC at 22 °C could be explained by the prevalence of a waterborne psychrophilic flora in the biofilm.

The greater frequency and higher concentrations of PA detected in the water dispensed from MWDs as compared to the input water confirms the microorganism’s ability to colonize the circuits of these devices [6,7]; since PA is naturally present in water and has low nutritional needs, it represents a typical microorganism of biofilms [19]. PA infections are an important cause of morbidity and mortality in immunocompromised patients [14], and tap water appears to be a significant route of transmission in hospitals. While the risk of colonization from ingesting contaminated drinking water is generally low, the risk becomes slightly higher if the subject is taking an antibiotic resisted by *P. aeruginosa* [19]. Skin exposure and inhaling aerosol appear to carry the greatest health risk [14].

Other species of NF-GNB detected in our study are commonly associated with various serious infections in healthcare settings. *S. maltophilia* is a waterborne emerging multidrug-resistant opportunistic pathogen responsible for hospital and community-acquired infections, particularly in immunocompromised patient populations [20], and has been reported to be transmitted through tap water [21,22,23]. A significant feature of *S. maltophilia* is its ability to form biofilms on surfaces, such as plastics [20], and it is consequently able to colonize the circuits of MWDs and to reach concentrations to 10^3^ cfu/250mL in the output water.

Particularly interesting was the detection, in one sample of RO-treated water, of *B. pseudomallei*, a pathogen for which there is evidence of high health significance related to its occurrence in drinking water [14]. The organism occurs predominantly in tropical regions and may multiply in water supplies; the number of organisms in drinking water that would constitute a significant risk of infection is not known [14].

Other NF-GNBs found in our study that are a potential risk for infections in immunocompromised subjects are *D. acidovorans* [24,25], *P. putida* [26] and *P. fluorescens*. The latter species was responsible for an outbreak due to contaminated drinking water in a bone marrow transplant unit following the pharyngeal colonization in hematological patients, ascertained through genotype analysis of the isolated strains [27].

The presence of *S. aureus* in one sample of municipal tap water is almost certainly the result of contamination of the outlet tap by the users. *S. aureus* is a common member of the human microflora and can be released into drinking water supplies by human contact. However, there is no evidence of transmission through the consumption of drinking water [14]. Furthermore, the presence of CoNS, normal inhabitants of human skin and mucous membranes, is the outcome of contamination of human origin. CoNS are becoming increasingly important as causes of hospital or healthcare-related infections, in particular *S. saprophyticus* [28]. In our study, the potentially harmful staphylococci species did not show the capacity to colonize the POU devices, since their concentrations were very low also in the output water.

From a chemical point of view, microfiltration does not appear to modify the mineral content of the input water, whereas treatment with reverse osmosis removes almost all of the minerals. The import role of water in supplying minerals like calcium and magnesium is well known [29], especially in certain groups of the population, such as the elderly [30]. Although the main source of these minerals is food, water consumption may represent a low, but constant and long-term source of calcium and magnesium. These minerals may, in fact, be absorbed better from water than from food, as they exist in a chemical form easily available for uptake and are usually ingested in small doses throughout the day [31,32].

Reductions in the intake of waterborne Mg and Ca (and therefore, in hardness) could potentially increase the risks for cardiac abnormalities and other pathologies [33,34]. A meta-analysis of case-control and cohort studies examining the possible relationship of water hardness to cardiovascular mortality concluded that the concentration of Mg in drinking water appears to be inversely related to cardiovascular mortality [35].

The WHO believes that consumers of drinking water dispensed by RO devices should be made aware of the changes in mineral composition that arise and the possible consequences for their health [29]. Additionally, this suggests that manufacturers of these devices should provide a bypass for a suitable portion of municipal tap water in order to maintain a certain level of minerals in the water consumed [29]. In our study, although all of the ROWDs were fitted with a bypass valve, this function, which would allow a blending between the permeate and municipal tap water to regulate the saline level, was not activated in any of the units examined.

With regards to sodium, the Italian guidelines for a healthy diet [36] recommend reducing the daily intake of this mineral, especially in advanced age. However, the contribution from drinking water is generally small, and in our study, the samples of municipal tap water entering the devices contained quite low levels of sodium, much lower than those normally found in food. Therefore, the reverse osmosis treatment had no significant impact on sodium daily intake.

In summary, since reverse osmosis is not particularly advantageous from a nutritional point of view, ROWDs should be used only when it is opportune to remove organic and inorganic chemicals of potential concern, such as arsenic, lead, copper, chromium and cadmium (e.g., in particular situations where metals may be released from old plumbing lines in long-standing buildings).

## 5. Conclusions

This report highlights a worsening of the bacteriological quality and, in the case of reverse osmosis treatment, also of the nutritional quality of the drinking water treated at the point of use consumed in healthcare facilities for the elderly.

To minimize bacterial growth, it is important to perform an adequate and continuous maintenance of the devices, and in particular, it is essential to ensure the efficacy and frequency of disinfection procedures, especially if the users are subjects at risk. Furthermore**,** the water dispensed by POU devices should undergo careful periodic bacteriological monitoring, which, in addition to the common bacteriological parameters required for drinking water, also involves the determination of potentially pathogenic non-enterobacterial species.

Finally, before installing a reverse osmosis POU treatment device, it is important to know the original characteristics of the tap water and to find a balance between the potential benefits of this device and the potential negative effect of reducing the calcium and magnesium levels.

In conclusion, the use of POU devices in healthcare facilities for the elderly is not particularly recommended, especially if the devices are not continuously and correctly maintained by specifically qualified staff.
